# The Parasite-Derived Peptide, FhHDM-1, Selectively Modulates miRNA Expression in *β*-Cells to Prevent Apoptotic Pathways Induced by Proinflammatory Cytokines

**DOI:** 10.1155/2024/8555211

**Published:** 2024-07-10

**Authors:** Inah Camaya, Meredith Hill, Dayna Sais, Nham Tran, Bronwyn O'Brien, Sheila Donnelly

**Affiliations:** ^1^ The School of Life Sciences University of Technology Sydney, Ultimo, New South Wales, Australia; ^2^ School of Biomedical Engineering Faculty of Engineering and Information Technology University of Technology Sydney, Ultimo, New South Wales, Australia

**Keywords:** *β*-cell survival, FhHDM-1, IGF-2, miR-466i-5p, PI3K/Akt, type 1 diabetes

## Abstract

We have previously identified a parasite-derived peptide, FhHDM-1, that prevented the progression of diabetes in nonobese diabetic (NOD) mice. Disease prevention was mediated by the activation of the PI3K/Akt pathway to promote *β*-cell survival and metabolism without inducing proliferation. To determine the molecular mechanisms driving the antidiabetogenic effects of FhHDM-1, miRNA:mRNA interactions and *in silico* predictions of the gene networks were characterised in *β*-cells, which were exposed to the proinflammatory cytokines that mediate *β*-cell destruction in Type 1 diabetes (T1D), in the presence and absence of FhHDM-1. The predicted gene targets of miRNAs differentially regulated by FhHDM-1 mapped to the biological pathways that regulate *β*-cell biology. Six miRNAs were identified as important nodes in the regulation of PI3K/Akt signaling. Additionally, IGF-2 was identified as a miRNA gene target that mediated the beneficial effects of FhHDM-1 on *β*-cells. The findings provide a putative mechanism by which FhHDM-1 positively impacts *β*-cells to permanently prevent diabetes. As *β*-cell death/dysfunction underlies diabetes development, FhHDM-1 opens new therapeutic avenues.

## 1. Introduction

Type 1 diabetes (T1D) is attributable to an absolute lack of insulin due to the autoimmune-mediated destruction of the pancreatic beta (*β*) cells, with hyperglycaemia presenting when more than 90% of the *β*-cells are lost [1]. To date, strategies to suppress the inflammatory processes that destroy the *β*-cells have been unsuccessful, or at best transient. This outcome has been largely attributable to the lack of specificity of most immunosuppressive regimes and their associated side effects, which increase morbidity and induce further *β*-cell death. Therefore, the therapeutic focus has shifted to the *β*-cell because if these central cells can be rendered resistant to the proinflammatory intraislet milieu, then T1D could be prevented.

We have previously identified a novel parasite-derived molecule, termed FhHDM-1, which prevents T1D in nonobese diabetic (NOD) mice when delivered over a short time course (six intraperitoneal injections on alternate days) at a timepoint coincident with the initiation of autoimmunity [2]. Analyses showed that FhHDM-1 localised to the pancreas, where it interacted with *β*-cells, and this was associated with the preservation of *β*-cell mass [3]. In vitro, the treatment of *β*-cells (murine and human) with FhHDM-1 enhanced their survival/function under basal conditions and prevented apoptosis mediated by the proinflammatory cytokines (IL-1*β*, TNF, and IFN*γ*), which destroy *β*-cells during T1D pathogenesis. The treatment of *β*-cells with FhHDM-1, both in vivo and in vitro, activated the PI3K/Akt signaling pathway, which enhanced *β*-cell function and survival [3]. Importantly, unlike many PI3K/Akt pathway agonists, such as GLP-1 [4], FhHDM-1 promoted *β*-cell survival without inducing proliferation.

It has been well established that PI3K/Akt signaling is a major regulator of cell survival, proliferation, and metabolism [5, 6]. As such, this pathway represents a promising therapeutic target to enhance *β*-cell function and survival. A growing body of evidence shows that activation of PI3K/Akt signaling can directly and indirectly promote the survival and function of pancreatic *β*-cells [7–13]. However, it will be important to identify the specific PI3K/Akt pathway modulators that enhance *β*-cell function and prevent *β*-cell death without inducing *β*-cell proliferation [14]. Accordingly, FhHDM-1 has therapeutic potential to promote *β*-cell survival/function and prevent diabetes. However, the molecular mechanisms that are driving the activation of PI3K/Akt by this parasite-derived peptide are not yet understood.

FhHDM-1–treated *β*-cells had increased protein expression levels of the insulin growth factor 1 receptor (IGF1R) [3], which is an upstream activating receptor for the PI3K/Akt pathway [15]. This suggests that FhHDM-1 can modulate IGF1R levels to initiate PI3K/AKT pathway signaling, resulting in enhanced *β*-cell survival/function. Considering the central role of micro(mi)RNAs in the regulation of gene expression at the posttranscriptional level, the possibility that FhHDM-1 was influencing miRNA:mRNA networks within *β*-cells to increase the production of IGF1R and consequently activate the PI3K/Akt pathway to prevent cytokine-induced *β*-cell death was investigated.

miRNAs are short, single-stranded noncoding RNAs that regulate gene expression posttranscriptionally and, as such, have a central role in the regulation of cellular responses to both physiological and disease conditions [16]. More specifically, several miRNAs have been identified as key regulators of stress signals and the PI3K/Akt pathway, to exert positive effects on *β*-cell function and survival [17–25]. Therefore, to determine if alterations to the crosstalk between miRNAs and the PI3K/Akt pathway were mediating the beneficial effects of FhHDM-1, expression profiles for both miRNAs and mRNA were determined from *β*-cells, which were exposed to the proinflammatory cytokines that drive T1D pathogenesis in the presence and absence of FhHDM-1. The miRNAs that were differentially expressed (DE) were selected, and their corresponding gene targets (mRNA) were identified. These interactions revealed molecular networks that were regulated by FhHDM-1 to prevent cytokine-induced *β*-cell death and dysfunction, thus providing new insights into the positive cellular changes within *β*-cells induced by FhHDM-1.

## 2. Materials and Methods

### 2.1. Peptide Synthesis

A synthetic peptide corresponding to the sequence of the mature full-length native FhHDM-1 [26] was synthesised to 95% purity and determined to be endotoxin-free (GLBiochem, China). Previous analysis by CD spectroscopy verified that the structure of the synthetic peptide corresponded to that of the native peptide [2, 5].

### 2.2. *β*-Cell Culture, Sample Preparation, and RNA Extraction

The NIT-1 *β*-cell line established from the insulinomas that developed in a 10-week-old female NOD mouse [27] was used for in vitro analyses. NIT-1 cells (2 × 10^6^) were allowed to adhere overnight (37°C/5% CO_2_) before being left untreated or pretreated with vehicle control or FhHDM-1 (10 *μ*M) for 1 h prior to exposure to proinflammatory cytokines (10 ng/mL IL-1*β*, 50 ng/mL TNF, 50 ng/mL IFN*γ*; BD Pharmingen) for 24 h. Cells were then washed twice with sterile PBS, and total RNA was isolated using the isolate II RNA mini kit (Bioline, BIO52073), according to the manufacturer's instructions.

### 2.3. Sequencing of the miRNA and mRNA Content of *β*-Cells

The extracted RNA from three biological replicates was pooled to obtain sufficient material for sequencing. In total, three independent sets of pooled samples from each treatment were sequenced. The preparation of miRNA for sequencing was performed by Macrogen (Seoul) using a NEBNext Small RNA Library Sample Prep Kit for Illumina sequencing (Version 4.0). From the resulting fastq sequencing files (FastQC version 0.11.7), adaptor sequences were excised and filtered for low-quality (< 20 phred score) sequences and low-length sequences (< 17 bp) using the bioinformatic tool CutAdapt (v2.8). Final processed reads were sequentially aligned to the reference genome (mm10), miRbase (release 22.1), and noncoding RNA database, RNA central (version 14.0), to classify known miRNAs and other types of RNA such as tRNA and snRNA. A novel miRNA prediction was performed by miRDeep2 (version 2.0.0.8).

Sequencing of the transcriptome was performed on 18 paired-end samples by Macrogen (Seoul) using a TruSeq Stranded mRNA LT Sample Prep Kit (Illumina, part #15031047) and a NovaSeq 6000 S4 reagent kit (Illumina). The quality of the produced data was assessed by the phred quality score at each cycle using FastQC (version 0.11.7). Then, low-quality reads, contaminant DNA sequences, and PCR duplicates were removed to minimise biases in the analysis. Trimmed reads were mapped to the reference genome (mm10) with HISAT2 (version 2.1.0), a splice-aware aligner. Transcripts were assembled using StringTie (version 2.1.3b) with aligned reads. Expression profiles were calculated for each sample and transcript/gene as read count and fragments per kilobase of transcript per million mapped reads (FPKM).

### 2.4. Analysis of the Differential Expression of miRNAs and mRNAs

The raw read counts of miRNAs and mRNAs for each sample were analysed for differential expression using the DESeq2 package (v1.38.3) in Rstudio (Rstudio Team, v1.3.1073) to identify DE miRNAs in (i) untreated (Un) cells versus cells exposed to proinflammatory cytokines (CM), (ii) FhHDM-1 treated cells exposed to proinflammatory cytokines (FhHDM-1-CM) versus cells exposed to proinflammatory cytokines only (CM), and (iii) FhHDM-1-CM versus Un cells. The RNAs with a Hochberg adjusted *p* value < 0.1 were considered DE. All DE miRNAs were plotted using RPM values on a heat map and log-adjusted *p* value versus log2 fold change on volcano plots. A log2 fold change threshold of ±1 was then applied to the resulting list of DE miRNAs for consideration of potential roles in diabetes or *β*-cell biology.

### 2.5. Gene Target Prediction and Transcriptome Correlation

To determine the putative gene targets of all DE miRNAs (without log2 fold change cut-offs), three online target prediction tools were used: miRDB, DIANA, and TargetScan. For miRDB (https://mirdb.org/), only genes with a target score of > 80 were included, while miRNAs with more than 5000 predicted targets were excluded. For DIANA (http://diana.imis.athena-innovation.gr/DianaTools/index.php), the microT-CDS v5.0 database was used with the default threshold of 0.7. Lastly, a custom prediction with a murine background using the TargetScan database (http://www.targetscan.org) was completed. Only genes that were identified by all three tools were considered for further analysis. The combination of multiple predictive tools is one of the most effective approaches to yielding a subset of high-quality predictions with minimal false positives and providing the greatest effect in supporting the validation of gene targets. The resulting list of common predicted gene targets was then analysed using the Database for Annotation, Visualization, and Integrated Discovery (DAVID) v6.8 (https://david.ncifcrf.gov/home.jsp) functional annotation analysis tool to determine the enrichment of Kyoto Encyclopaedia of Genes and Genomes (KEGG) pathways. Where there were insufficient genes to attribute to pathways, gene ontology enrichment for molecular function and biological process was completed using Panther DB (http://www.pantherdb.org/). For the final analysis, the common predicted gene targets were correlated to DE genes from the same treatment groups within the NIT-1 *β*-cell transcriptome. The transcriptome-matched genes were similarly mapped to KEGG pathways. Cytoscape 3.9.1 was used to visualise miRNA-mRNA gene target interactions.

## 3. Results

### 3.1. FhHDM-1 Altered the Expression Levels of miRNAs in *β*-Cells Treated With Proinflammatory Cytokines

**Figure 1:**
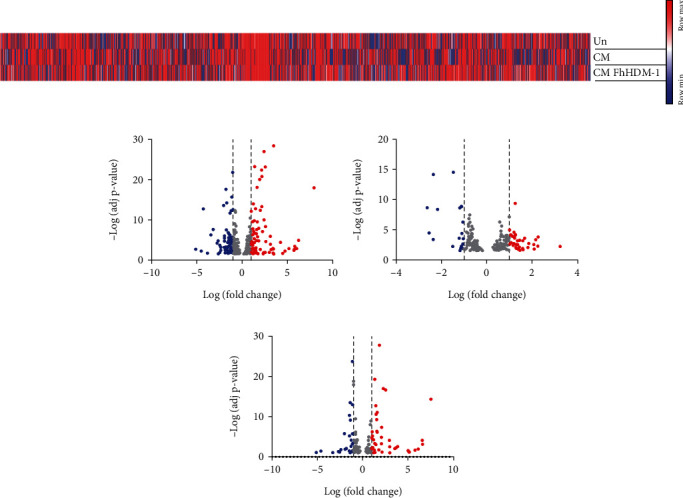
Exposure of *β*-cells to proinflammatory cytokines induced changes in miRNA expression levels, which were further altered by treatment with FhHDM-1. NIT-1 *β*-cells (2 × 10^6^) were left untreated (Un) or treated with FhHDM-1 (10 *μ*M, 1 h) prior to exposure to proinflammatory cytokines (CM: IL-1*β*, TNF, and IFN*γ*) for 24 h, followed by RNA extraction and sequencing. The raw read counts were analysed for differential expression (DE) using DESeq2 (version 1.38.3), with RNAs with a Hochberg adjusted *p* value < 0.1 considered as DE. (a) Heat map of relative abundance of miRNAs from *β*-cells (reads per million). (b–d) Volcano plot of all differentially expressed miRNAs. Dashed lines represent Log2FC ± 1; DE miRNAs below this threshold are represented in grey, and DE miRNAs beyond this threshold are shown in red (> 1) or blue (< 1). (b) *β*-Cells exposed to CM as compared to untreated cells had 291 DE miRNA beyond the threshold (77 up, 69 down); (c) FhHDM-1–treated *β*-cells as compared to *β*-cells exposed to CM alone showed 188 DE miRNA beyond the threshold (37 up, 19 down); (d) *β*-cells exposed to CM and treated with FhHDM-1 as compared to untreated cells had 144 DE miRNAs beyond the threshold (43 up, 25 down).

We have previously shown that FhHDM-1 prevented *β*-cell apoptosis driven by the same cocktail of proinflammatory cytokines that destroys *β*-cells during T1D pathogenesis [3]. Here, the molecular changes induced by FhHDM-1 treatment of *β*-cells under these same experimental conditions were investigated. Thus, NIT-1 *β*-cells were pretreated with vehicle control or FhHDM-1 (10 *μ*M) for 1 h before exposure to a combination of proinflammatory cytokines (10 ng/mL IL-1*β*, 50 ng/mL TNF, and 50 ng/mL IFN*γ*) for a further 24 h. RNA was then isolated for in-depth small RNA sequencing. Subsequent analyses identified the DE miRNAs in each treatment group, with RNAs with a Hochberg adjusted *p* value < 0.1 considered DE. In *β*-cells exposed to proinflammatory cytokines for 24 h, a total of 291 miRNAs were DE, as compared to Un controls (Figures 1(a) and 1(b), Table S1), thereby corroborating reports of substantial changes in the miRNA profile in the presence of proinflammatory cytokines [28]. Compared to *β*-cells treated with cytokines alone, the addition of FhHDM-1 reduced the total number of DE miRNAs to 188 miRNAs (Figures 1(a) and 1(c), Table S1). Of these, a total of 56 miRNAs (Figure 1(c)) reached beyond a log2 fold change threshold of ±1, with 37 upregulated and 19 downregulated.

By comparing the miRNA profile of *β*-cells exposed to both proinflammatory cytokines and FhHDM-1 to that of Un *β*-cells (Figure 1(d), Table S2), it was evident that FhHDM-1 was not simply blocking the impact of cytokine challenge on *β*-cells. Of the 144 DE miRNAs in these *β*-cells, the majority were also identified as being altered in response to proinflammatory cytokines alone, as compared to Un *β*-cells (Figure 1(b)). Furthermore, the fold change in expression of 46 of the upregulated miRNAs and 32 of the downregulated miRNAs identified in the FhHDM-1-treated, cytokine-challenged *β*-cells (as compared to cytokine treatment only) was almost identical to that observed in the cytokine-treated *β*-cells (as compared to Un).

This suggests that the presence of FhHDM-1 per se exerted a negligible effect on the altered expression of these miRNAs mediated by pro-inflammatory cytokines. However, comparing the 27 miRNAs that were identified as DE in all *β*-cell treatments (Figure 1, Table S2) revealed that FhHDM-1 generally reversed the cytokine effects on the expression levels of these specific miRNAs. The 13 miRNAs that had increased expression in *β*-cells challenged with proinflammatory cytokines as compared to Un cells had a lower fold change or a decrease in expression levels when FhHDM-1 was present (as compared to cytokines only or Un cells). The same effect was also evident for 11 miRNAs that were downregulated in response to cytokine treatment, with the addition of FhHDM-1 causing a less pronounced reduction in fold change or an increase in expression levels. These data supported the hypothesis that FhHDM-1 was mediating its beneficial effects on *β*-cells by modulating specific molecular targets. To next gain specific insights into the biological processes likely impacted by the altered miRNA expression levels in *β*-cells induced by FhHDM-1 treatment, analyses of the predicted gene targets for up- and downregulated miRNAs were then performed.

### 3.2. Predicted Gene Targets for DE miRNAs in FhHDM-1–Treated *β*-Cells Mapped to Metabolic and PI3K/Akt Signaling Pathways

To elucidate the functional roles of the DE miRNAs in regulating the biological activity of *β*-cells in the presence of FhHDM-1, gene targets for each miRNA were predicted from the 3′UTR regions of genes within the murine genome using the software tools miRDB, DIANA tools, and TargetScan (Table S3). To obtain high-quality predictions, limit false positives, and identify the most authentic miRNA:mRNA relationships, only those gene targets that were identified by *all* three tools were selected for further analysis. With these parameters, a total of 324,884 predicted gene targets were identified, of which 321,788 and 3096 were attributed to upregulated or downregulated miRNAs, respectively, in FhHDM-1–treated *β*-cells in the presence of proinflammatory cytokines (Table S4).

**Figure 2:**
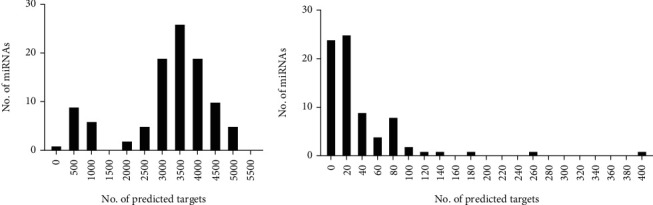
Treatment of *β*-cells with FhHDM-1 modulated miRNA expression. (a) Upregulated and (b) downregulated miRNAs in FhHDM-1–treated *β*-cells under proinflammatory cytokine conditions, with their total number of predicted gene targets that were common across all three prediction tools (miRDB, DIANA, and TargetScan) used.

The frequency distribution of the number of targets per miRNA (Figure 2) showed that within the upregulated miRNA list, gene targets were evenly distributed across miRNAs, with most having 3000–4500 gene targets. The miRNAs with the most predicted gene targets included miR-128-3p with 4788, miR-494-3p with 4820, miR-329-3p with 4892, miR-27b-3p with 5029, and miR-124-3p with 5056. In contrast, most downregulated miRNAs had between one and 80 predicted gene targets, except for some miRNAs that had greater than 100 targets. These included miR-330-3p with 105 gene targets, miR-129-5p with 117, miR-350-5p with 140, miR-493-5p with 185, miR-181a-5p with 250, and miR-466i-4p with 394.

**Figure 3:**
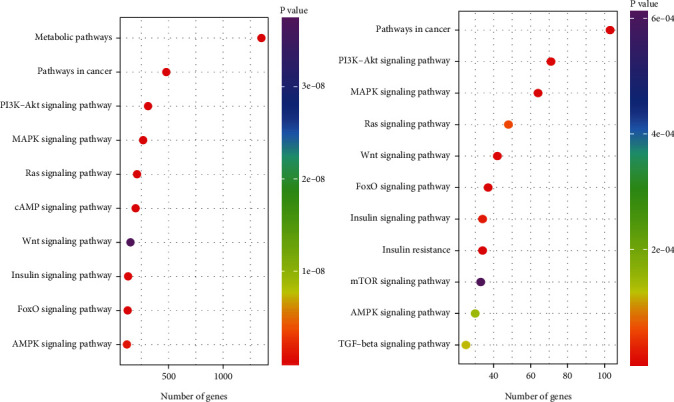
KEGG pathway analysis of predicted gene targets from (a) upregulated and (b) downregulated miRNAs in FhHDM-1–treated *β*-cells revealed highly represented pathways associated with *β*-cell biology. Dot plot showing the Top 10 pathways of most likely relevance to *β*-cell survival and function. The genes in each pathway are reported in Tables S4 and S5. Dot colour indicates *p* value, and dot placement on the *x*-axis indicates the number of genes in that pathway.

These predicted gene targets were then mapped to biological pathways using KEGG pathway analysis, resulting in the identification of 269 (Table S5) and 142 (Table S6) biological pathways, respectively, for the miRNAs that were increased and decreased in expression. An inverse relationship between the expression of a given miRNA and its gene target(s) was presumed. Therefore, the gene targets for the miRNA sequences that were reduced in expression levels by FhHDM-1 treatment of *β*-cells were predicted to exhibit increased expression levels, thereby contributing to the increased survival of *β*-cells mediated by FhHDM-1, and vice versa. The predicted gene targets of miRNAs from FhHDM-1–treated *β*-cells exposed to proinflammatory cytokines mapped to biological pathways that were intricately linked to *β*-cell biology. The following signaling pathways were shared between the upregulated (Figure 3(a)) and downregulated (Figure 3(b)) miRNA gene targets: PI3K/Akt, MAPK, Ras, Wnt, FoxO, AMPK, insulin signaling, and those involved in cancer. Unique pathways to which upregulated miRNA gene targets were attributed included metabolic pathways and cAMP signaling. On the other hand, unique pathways to which downregulated miRNA gene targets were mapped included insulin resistance, mTOR, and TGF-*β* signaling.

Since PI3K was shared between upregulated and downregulated miRNA gene targets, it was difficult to delineate the bona fide effect(s) of FhHDM-1 by these analyses. Therefore, the specific genes involved in the PI3K/Akt pathway, as predicted by the DAVID (v6.8) functional annotation analysis tool (https://david.ncifcrf.gov), were examined further. For the downregulated gene targets (Figure S1), most (if not all) genes were predictively affected, indicating that the PI3K pathway was broadly impacted by FhHDM-1, though no specific effect could be ascertained through these analyses. The regulation of these genes within the PI3K pathway was evenly distributed across 6354 different miRNAs, though the topmost functional candidates were miR-128-3p, miR-124-3p, and miR-107-3p, which govern 104, 101, and 100 genes, respectively. Among the upregulated gene targets (Figure S2), IGF and its downstream targets, such as IRS1, Ras, and ERK, were predictively increased in expression. Importantly, while PI3K was activated, its inhibitor PTEN was not. Of the 94 genes that were predicted to be increased within the pathway, regulation was mostly attributed to three miRNAs, miR-466i-5p, miR-3473a, and miR-350-5p, which govern 10, 8, and 7 genes, respectively. The activation of these upregulated gene targets, through downregulation of the corresponding regulator miRNA(s), ultimately exerts prosurvival effects. This finding corroborates the outcomes observed for FhHDM-1–treated *β*-cells, in which *β*-cell survival and metabolism were enhanced and apoptosis was inhibited [3].

### 3.3. Comparing Predicted Gene Targets to DE mRNAs in *β*-Cells Identified the miRNA:mRNA Interactions Altered by FhHDM-1 to Activate PI3K/Akt Signaling Pathways and Enhance Metabolic Activity

To more precisely determine the relationship between the DE miRNAs and their target genes in FhHDM-1–treated *β*-cells under proinflammatory conditions, the common predicted gene targets of miRNAs (Table S4) were matched to gene expression level changes identified from transcriptome data of FhHDM-1–treated *β*-cells (Table S7). The resulting genes (Table S8) were then mapped to KEGG pathways, with 37 (Table S9) and 14 (Table S10) pathways associated with the downregulated and upregulated genes, respectively.

**Figure 4:**
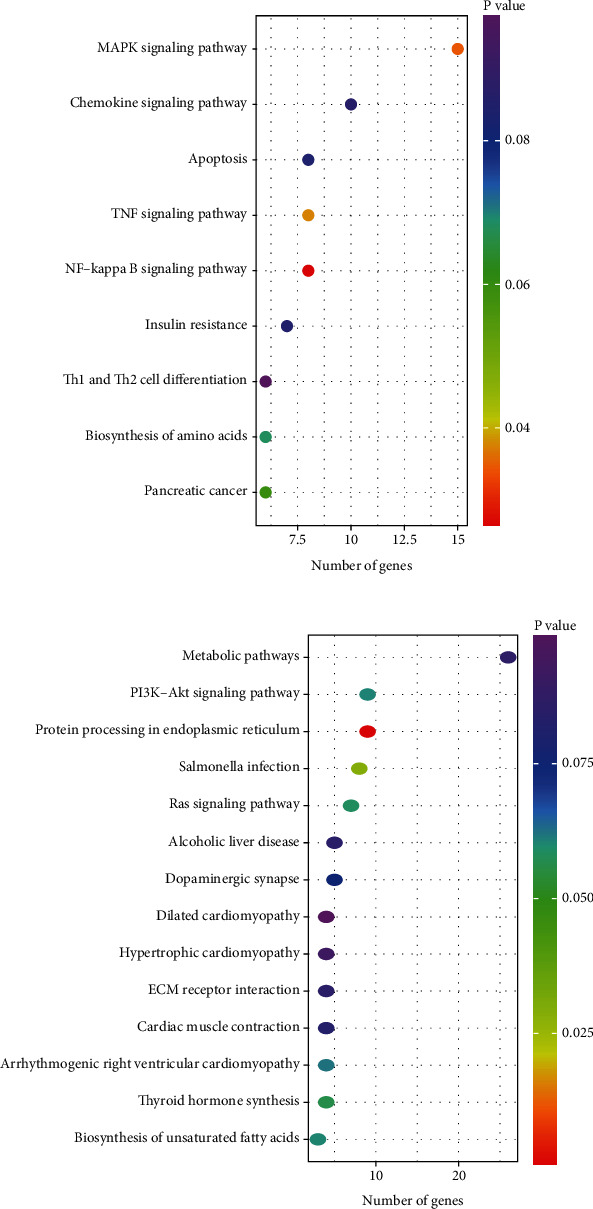
KEGG pathway analysis of matched gene targets from (a) upregulated or (b) downregulated miRNAs in FhHDM-1–treated *β*-cells yielded pathways of specific relevance to *β*-cell biology. Dot plot showing all pathways identified from KEGG pathway analysis. The genes in each pathway are shown in Tables S8 and S9. Dot colour indicates *p* value, and dot placement on the *x*-axis indicates the number of genes in that pathway.

Of the 37 pathways associated with downregulated genes, the most relevant pathways to *β*-cell biology and/or diabetes (Figure 4(a)) included MAPK signaling, apoptosis, TNF signaling, NFK*β* signaling, insulin resistance, and pancreatic cancer. This suggests that FhHDM-1 downregulated apoptotic pathways, pathways associated with inflammation (i.e., TNF and insulin resistance), and more specifically, inflammatory pathways associated with diabetes. Furthermore, the predicted downregulation of pancreatic cancer pathways corroborates previous observations that FhHDM-1 selectively promotes *β*-cell survival but not proliferation [3].

On the other hand, the upregulated genes were highly attributed to metabolic pathways and PI3K/Akt signaling, with 26 and nine genes identified, respectively (Figure 4(b)). The metabolic pathways fell into the general categories of energy, carbohydrate, lipid, amino acid, and nucleotide metabolism and featured pathways such as the citrate cycle, glycolysis/gluconeogenesis, fatty acid biosynthesis/degradation, fructose/mannose metabolism, phosphonate/phosphinate metabolism, and nicotinate/nicotinamide metabolism. Of the genes attributed to the PI3K/Akt signaling pathway, IGF-2, a ligand that induces expression and activation of the IGF1R to promote *β*-cell survival, was identified [29].

### 3.4. The miRNA:mRNA Interactome in FhHDM-1–Treated *β*-Cells Characterises the Molecular Networks That Regulate PI3K Signaling and Metabolic Pathways to Counteract the Effects of Proinflammatory Cytokines

**Figure 5:**
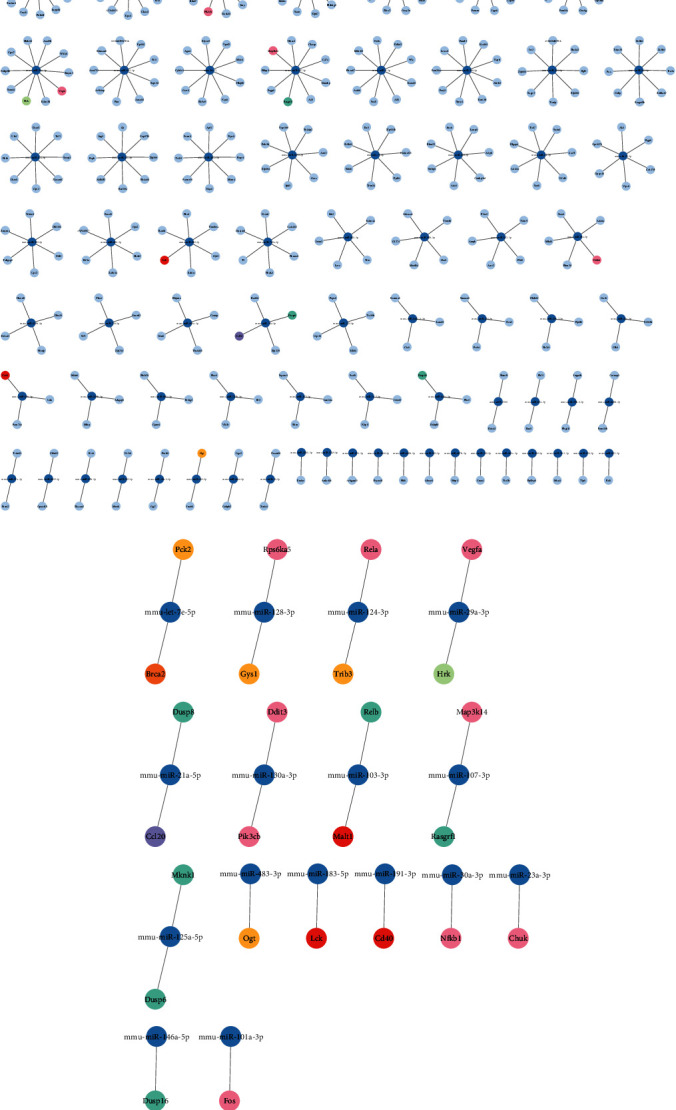
Interaction network of transcriptome-matched genes downregulated in the transcriptome with their corresponding regulatory miRNA upregulated in FhHDM-1–treated *β*-cells under proinflammatory conditions. The transcriptome-matched genes were attributed to KEGG pathways, after which the genes within the topmost relevant biological pathways were matched back to their corresponding regulatory miRNA to generate interaction networks displaying (a) all interactions or (b) only the interactions within the relevant pathways. Each dark blue node represents the miRNAs, and connections to light blue nodes represent the corresponding matched gene target(s), apart from gene nodes attributed to relevant biological pathways, namely, apoptosis, insulin resistance, MAPK, metabolic pathways, NF*κ*B, PI3K, pancreatic cancer, and TNF, which are colour coded according to the legend. Genes that appear across multiple pathways are coloured pink. Created using Cytoscape 3.9.1.

Interaction networks were built from the transcriptome-matched genes and their corresponding regulatory miRNAs to identify candidate miRNAs that were central players in the biological effects mediated by FhHDM-1. The genes predicted to be downregulated (due to a corresponding upregulation of cognate miRNAs) were clustered discretely (Figure 5), with each miRNA regulating unique and distinct sets of gene targets. The genes that were attributed to significant KEGG pathways, including apoptosis, insulin resistance, TNF, NFKB, pancreatic cancer, and MAPK, were distributed among different miRNAs. Consequently, no individual miRNA was predicted to exert a broad regulatory effect, which could be singularly attributed to the enhanced viability of *β*-cells mediated by FhHDM-1. However, based on the number of gene targets per miRNA, let-7e-5p, miR-124-3p, and miR-101a-3p were the most impactful, regulating 51, 47, and 43 gene targets, respectively. These three miRNAs regulate genes associated with pancreatic cancer (Bcra2) and insulin resistance (Pck2 and Trib3) and regulate the expression levels of genes (Rela and Fos) among shared pathways.

**Figure 6:**
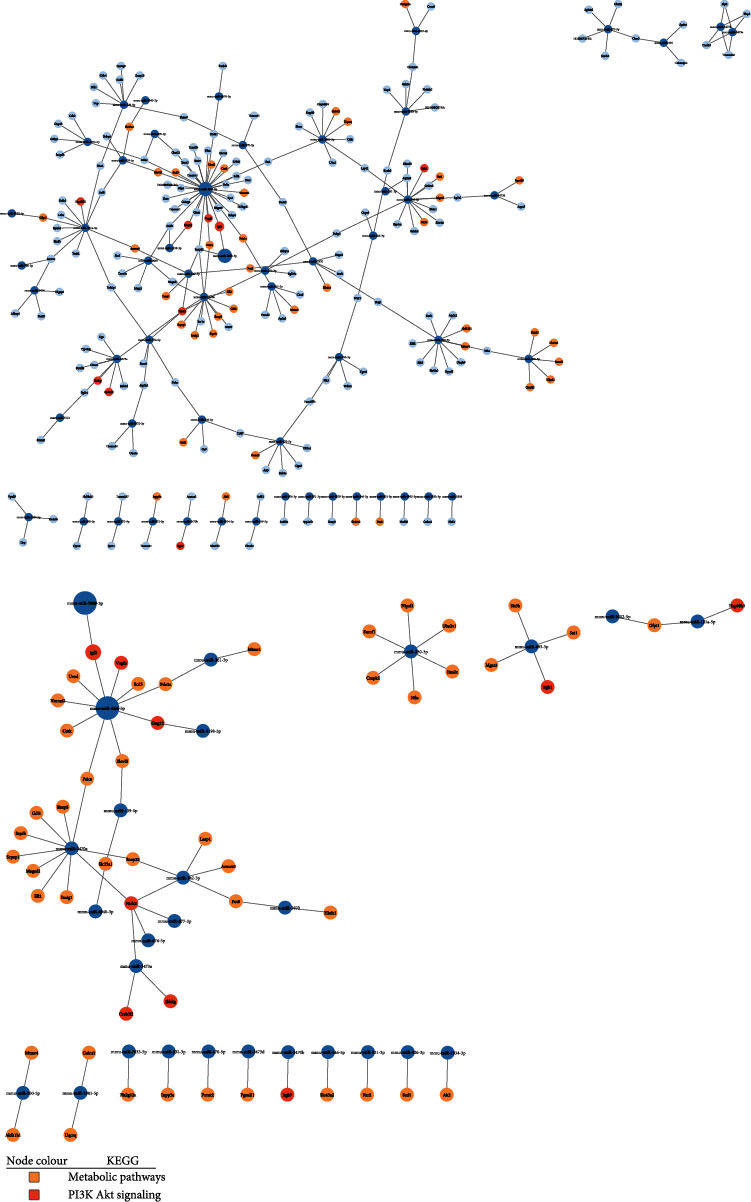
Interaction network of matched genes upregulated in the transcriptome with their corresponding regulatory miRNA downregulated in FhHDM-1–treated *β*-cells under proinflammatory conditions. The transcriptome-matched genes were attributed to KEGG pathways, after which the genes within the topmost relevant biological pathways were matched back to their corresponding regulatory miRNA to generate interaction networks displaying (a) all interactions or (b) only the interactions within the relevant pathways. Each dark blue node represents the miRNAs, and connections to light blue nodes represent the corresponding matched gene target(s), apart from gene nodes attributed to metabolic pathways and PI3K, which are colour coded according to the legend. Created using Cytoscape 3.9.1.

Among the upregulated genes (i.e., correlated to downregulated miRNA expression levels), IGF-2 was of particular interest given its pivotal role in the activation of the PI3K pathway and the promotion of *β*-cell survival. Within the interaction network (Figure 6), IGF-2 was regulated by miR-466i-5p and miR-7689-3p, both of which also regulate other genes associated with PI3K signaling and metabolic pathways. Moreover, miR-466i-5p regulated the greatest number (*n* = 36) of genes, thus establishing itself as a miRNA of potential significance in the molecular mechanism of action by which FhHDM-1 preserves *β*-cells under proinflammatory conditions.

## 4. Discussion

It is widely accepted that miRNAs orchestrate critical alterations in gene expression levels to ensure optimal *β*-cell function and survival and dynamic adaptations to metabolic signals, notably changes to blood glucose concentrations [30, 31]. This fine-tuning of gene expression regulates the differentiation of *β*-cells and contributes to the maintenance of their unique phenotype, influencing their mass, metabolic activity, and survival in response to external cues and challenges. However, changes to this miRNA landscape result in *β*-cell dysfunction associated with the development of diabetes [32, 33]. Acknowledging the central role of miRNAs in disease development has driven efforts to identify pharmacological agents that can correct expression level imbalances, which are associated with the development of diabetes [31, 34–36].

We have previously identified a novel parasite-derived molecule, termed FhHDM-1, which prevented T1D development in NOD mice when delivered over only a short time course, coincident with the initiation of autoimmunity [2]. The FhHDM-1 peptide localised to the pancreas, interacted directly with *β*-cells, and activated the PI3K/Akt signaling pathway. Consequently, insulin production was increased, *β*-cell survival was enhanced, and apoptosis was prevented, even in the presence of the proinflammatory cytokines that destroy *β*-cells during T1D pathogenesis [3]. Here, we have now shown that these positive effects are associated with substantial changes to the miRNA:mRNA interaction networks within *β*-cells. Specifically, genes that were downregulated (because of upregulation of their cognate miRNAs) by FhHDM-1 treatment under proinflammatory conditions mapped to the biological pathways controlling apoptosis, insulin resistance, and inflammation. Moreover, the downregulation of genes associated with pancreatic cancer pathways suggests that the miRNAs modulated by FhHDM-1 may prevent *β*-cell proliferation, which could result in malignancy. This is also consistent with cellular studies using murine and human *β*-cells, showing that FhHDM-1 promoted *β*-cell viability/function without inducing proliferation [3]. Genes that were upregulated (due to downregulated cognate miRNAs) by FhHDM-1 treatment of *β*-cells mapped to the biological pathways of PI3K/Akt signaling and other associated prosurvival pathways. This finding corroborates our previous observations that FhHDM-1 activated the PI3K/Akt signaling pathway, thereby exerting antiapoptotic and anti-inflammatory effects [3].

A detailed examination of the specific genes targeted within the activated PI3K pathway of FhHDM-1–treated *β*-cells revealed a significant increase in IGF-2 expression levels. This finding is of mechanistic relevance as IGF-2 is a primary agonist of IGF1R, an upstream receptor that activates the PI3K/Akt pathway in *β*-cells. IGF-2 is highly expressed in primary islets and in purified *β*-cells and is secreted in response to glucose [37, 38]. The antiapoptotic effects associated with the autocrine actions of IGF-2 regulate *β*-cell function early in life, with impaired *β*-cell function in adult rodents linked to decreased IGF-2 expression levels within the pancreas during embryogenesis [39, 40]. Moreover, the neonatal phase of pancreatic remodelling characterised by waves of *β*-cell apoptosis coincides with reduced expression levels of IGF-2 within the islets [41]. Conversely, apoptosis can be prevented by increased circulating levels of IGF-2, as shown in models in which there was transgenic overexpression of IGF-2 in peripheral tissues [42]. In adult mice, transgenic expression of IGF-2 results in increased *β*-cell mass [43]. Further, *β*-cell injury triggers the reexpression of IGF-2, which is crucial for subsequent *β*-cell regeneration [44]. Thus, it is evident that autocrine IGF-2 is an important regulator of *β*-cell mass in both foetal and adult life. Furthermore, IGF dysregulation precedes and follows T1D development in humans [45]. Unsurprisingly, IGF-2 has been demonstrated to ameliorate disease in various models of diabetes [41, 46, 47]. The increase in IGF-2 expression levels in *β*-cells induced by FhHDM-1 in this study is comparable to levels reported after the treatment of *β*-cells with GLP-1. In this instance, GLP-1 activated the IGF-2/IGF1R autocrine loop and consequently the PI3K/Akt pathway to exert antiapoptotic effects [48]. While there is little sequence homology between FhHDM-1 and GLP-1, both form alpha helices, which may correlate with the activation of the IGF-2/IGF1R/PI3K signaling axis. However, unlike GLP-1, FhHDM-1 treatment of *β*-cells does not increase proliferation.

Although no singular miRNA was identified to mediate the FhHDM-1-induced changes to the *β*-cells, several miRNAs were deemed to be broadly influential due to their number of gene targets. Most notable among the upregulated miRNAs that governed common predicted gene targets within the PI3K pathway were miR-128-3p, miR-124-3p, and miR-107-3p. In addition, the analysis of transcriptome-matched gene targets of the upregulated miRNAs identified let-7e-5p, miR-124-3p, and miR-101a-3p as candidates of interest. Among the miRNAs that were downregulated in response to FhHDM-1 treatment of *β*-cells, the top miRNAs that regulated common predicted gene targets within the PI3K pathway included miR-466i-5p, miR-3473a, and miR-350-5p, whereas the most notable miRNAs from the final analysis of transcriptome-matched genes were miR-466i-5p and miR-7689-3p. Of these miRNAs, only miR-101a-3p, miR-124-3p, and let-7e-5p have been previously associated with the development of diabetes [49, 50].

Corroborating the proposed beneficial increase in expression induced by FhHDM-1, miR-124-3p was reportedly downregulated in plasma-derived extracellular vesicles of children with recent onset T1D [51], and let-7e-5p expression was reduced in the endocrine cells of mice on a high-fat diet presenting with glucose intolerance, akin to T2D [52]. However, contradicting the increased expression of miR-101a-3p induced by FhHDM-1, the abundance of this miRNA in sera was found to be three times higher in T1D patients in comparison to control groups [50], suggesting a potential role in the progression of disease (or as a marker of diabetes development) rather than a protective effect as predicted by the analysis presented here. Furthermore, exposure of a murine *β*-cell line (MIN6) to IL-1*β* resulted in an increase in the expression of miR-101a-3p, which was shown to mediate *β*-cell death by reducing the expression of the antiapoptotic protein Bcl2 [53]. These opposing effects demonstrate the complexities of miRNA expression and the regulation of gene targets. It is attractive to speculate that, in the context of diabetes development, FhHDM-1-induced changes to miRNA expression are dynamic, and it is the net balance between the expression of “beneficial” and “detrimental” miRNAs that ultimately skews the balance towards benefit, as evidenced by the prevention of *β*-cell death and diabetes. Further studies investigating the changes in miRNA expression levels throughout the process of diabetes development will validate this premise.

Among the miRNAs identified in this study, it has been previously reported that miR-128-3p (upregulated by FhHDM-1 treatment of *β*-cells) mediates antiapoptotic and anti-inflammatory effects in a range of cell types under a variety of environmental scenarios [54–56], while miR-466i-5p (downregulated by FhHDM-1) has been shown to promote the apoptosis of brain neurons [57]. Additionally, expression levels of miR-107-3p have been reportedly increased in rat-derived *β*-cells (INS1) following treatment with GLP-1, but no specific functional correlation has been investigated [58]. However, combined with the evidence that GLP-1 enhanced *β*-cell survival [48], it is tempting to speculate that this miRNA may be involved in the activation of prosurvival and antiapoptotic gene networks induced by GLP-1 and, as shown here, by FhHDM-1.

This study adopted a robust and three-pronged approach in which miRNAs were analysed with multiple gene target prediction tools, from which only the common gene targets were integrated with the mRNA transcriptome of the same *β*-cells. This approach enabled the most accurate identification of genes regulated by the DE miRNAs. In elucidating the biological outcomes of these miRNA:mRNA interactions, the broadly accepted inverse correlation between the expression of a given miRNA and the expression of its gene targets was assumed [59, 60]. However, the expression of certain miRNAs and their gene targets can also be positively correlated. Of interest in this study was miR-483-3p, which, although just below the Log2FC ± 1 threshold, was upregulated in FhHDM-1–treated *β*-cells (Table S1) and is reportedly coexpressed with IGF-2. Furthermore, there is a positive feedback loop whereby miR-483-5p enhances IGF-2 expression [61]. This suggests that increased expression levels of miR-483-3p, induced by FhHDM-1 treatment, might be driving increased production of IGF-2 and subsequent activation of the PI3K/Akt pathway, with consequent positive effects on *β*-cell survival and function.

As such, modulation of PI3K/Akt signaling by FhHDM-1 represents a promising therapeutic target for diabetes prevention. Activation of the pathway, singularly and/or through miRNA interactions, would regulate the *β*-cell processes that determine their fate. *β*-cell mass lost from immune destruction could be reduced or maintained by PI3K/Akt-mediated stimulation of *β*-cell survival, metabolic, and antiapoptotic pathways. While these pathways are central to the prevention of T1D, they also mitigate the development of T2D. Although their etiopathologies differ, T1D and T2D are characterised by a decline in *β*-cell mass and function largely due to a proinflammatory islet environment. In T2D, as insulin demands increase in response to insulin resistance, *β*-cells undergo compensatory hyperplasia, ultimately causing *β*-cell exhaustion and death [62, 63]. Low-grade islet inflammation and elevated amounts of proinflammatory cytokines during the development of T2D compromise *β*-cell function and survival [64]. Furthermore, genome-wide association studies of T2D show that most genes identified play roles in the modulation of *β*-cell mass and/or function [65–68]. Therapeutic strategies to enhance PI3K/Akt signaling in *β*-cells could also be beneficial for islet transplantation to counteract the *β*-cell death that occurs throughout the process of islet isolation and delivery and then posttransplant due to allogeneic and autoimmune destruction. Therefore, the alteration in the miRNA landscape by FhHDM-1, resultant activation of the PI3K/Akt pathway, and preservation of functional *β*-cell mass open therapeutic avenues for the prevention of T1D and T2D and treatment by enhancing islet transplantation outcomes.

## 5. Conclusion

This study has provided a detailed characterisation of miRNA and mRNA expression patterns and their associated co-networks in *β*-cells treated with FhHDM-1 and challenged with a cocktail of proinflammatory cytokines relevant to the inflammatory conditions underpinning T1D and T2D. Utilising an accepted bioinformatic pipeline, six miRNAs (miR-128-3p, miR-124-3p, miR-107-3p, miR-466i-5p, miR-3473a, and miR-350-5p) were identified as important nodes in the regulation of PI3K/Akt signaling in *β*-cells to enhance survival against immune-mediated cell death. In addition, IGF-2 was recognised as a possible activating ligand that mediated the beneficial effects of FhHDM-1 treatment of *β*-cells, with its expression predictably increased by several DE miRNAs. These findings establish a solid foundation for future targeted analyses to fully determine how the identified miRNAs and their corresponding gene targets (IGF-2 in particular) contribute to the positive effects of FhHDM-1 on the function and survival of *β*-cells.

## Data Availability

The datasets underlying this study are available from the corresponding author on reasonable request.

## Nomenclature

AMPK: AMP-activated protein kinase
CD: circular dichroism
CM: proinflammatory cytokine mix
DE: differentially expressed
FhHDM-1: *Fasciola hepatica* helminth defense molecule-1
FoxO: forkhead box O
GLP-1: glucagon-like peptide-1
IGF: insulin growth factor
IGF1R: insulin growth factor 1 receptor
IRS-1: insulin receptor substrate-1
MAPK: mitogen-activated protein kinase
NFKB: nuclear factor kappa-light-chain-enhancer
NOD: nonobese diabetic
PI3K/Akt: phosphoinositide 3-kinase/protein kinase B
PTEN: phosphatase and tensin homolog
STZ: streptozotocin
T1D: Type 1 diabetes
Un: untreated

## Supporting Information

Supporting Information 1 Figure S1. The (downregulated) gene targets of upregulated miRNA impacted within the PI3K/Akt pathway determined by DAVID (v6.8) functional annotation analysis tool. Figure S2. The (upregulated) gene targets of downregulated miRNAs impacted within the PI3K/Akt pathway as determined using DAVID (v6.8) functional annotation analysis tool.

Supporting Information 2 Table S1A. Differentially expressed miRNAs in FhHDM-1–treated *β*-cells exposed to proinflammatory cytokines, compared to cytokines alone. Table S1B. Differentially expressed miRNAs in untreated *β*-cells compared to *β*-cells exposed to proinflammatory cytokines. Table S1C. Differentially expressed miRNAs in untreated *β*-cells compared to FhHDM-1–treated *β*-cells exposed to proinflammatory cytokines.

Supporting Information 3 Table S2. Differentially expressed miRNAs common across all cell treatment conditions; *β*-cells exposed to proinflammatory cytokines (CM) compared to untreated cells (UT); FhHDM-1–treated *β*-cells challenged with cytokines (FhHDM-1-CM) compared to cytokines (CM) alone; FhHDM-1–treated *β*-cells challenged with cytokines (FhHDM-1-CM) compared to untreated cells (UT).

Supporting Information 4 Table S3. Predicted gene targets for differentially expressed miRNAs in FhHDM-1–treated *β*-cells exposed to proinflammatory cytokines compared to cytokines (CM) alone.

Supporting Information 5 Table S4. Predicted gene targets of differentially expressed miRNAs in FhHDM-1–treated *β*-cells exposed to proinflammatory cytokines compared to cytokines (CM) alone, common across all online miRNA gene target prediction tools mIRDB, DIANA, and TargetScan.

Supporting Information 6 Table S5. KEGG pathway analysis of predicted gene targets from miRNA upregulated in FhHDM-1–treated *β*-cells exposed to proinflammatory cytokines compared to cytokines alone.

Supporting Information 7 Table S6. KEGG pathway analysis of predicted gene targets from miRNA downregulated in FhHDM-1–treated *β*-cells exposed to proinflammatory cytokines compared to cytokines alone.

Supporting Information 8 Table S7. Differentially expressed genes in the transcriptome of FhHDM-1–treated *β*-cells exposed to proinflammatory cytokines compared to cytokines alone.

Supporting Information 9 Table S8. Matched gene targets common between transcriptome and predicted gene targets of differentially expressed miRNA in FhHDM-1–treated *β*-cells under proinflammatory conditions compared to proinflammatory cytokine only (CM) controls.

Supporting Information 10 Table S9. KEGG pathway analysis of matched genes downregulated in the transcriptome with their corresponding regulatory miRNA upregulated in FhHDM-1–treated *β*-cells exposed to proinflammatory cytokines compared to cytokines alone.

Supporting Information 11 Table S10. KEGG pathway analysis of matched genes upregulated in the transcriptome with their corresponding regulatory miRNA downregulated in FhHDM-1–treated *β*-cells exposed to proinflammatory cytokines compared to cytokines alone.
