# Supplementary material for: The Parasite-Derived Peptide, FhHDM-1, Selectively Modulates miRNA Expression in β-Cells to Prevent Apoptotic Pathways Induced by Proinflammatory Cytokines
Source: J Diabetes Res. 2024 Jul 10;2024:8555211. doi: 10.1155/2024/8555211 (PMC11254460; doi:10.1155/2024/8555211)
Supplement: Supporting Information 1 — Figure S1. The (downregulated) gene targets of upregulated miRNA impacted within the PI3K/Akt pathway determined by DAVID (v6.8) functional annotation analysis tool. Figure S2. The (upregulated) gene targets of downregulated miRNAs impacted within the PI3K/Akt pathway as determined using DAVID (v6.8) functional annotation analysis tool. [file 8555211.f1.pdf]

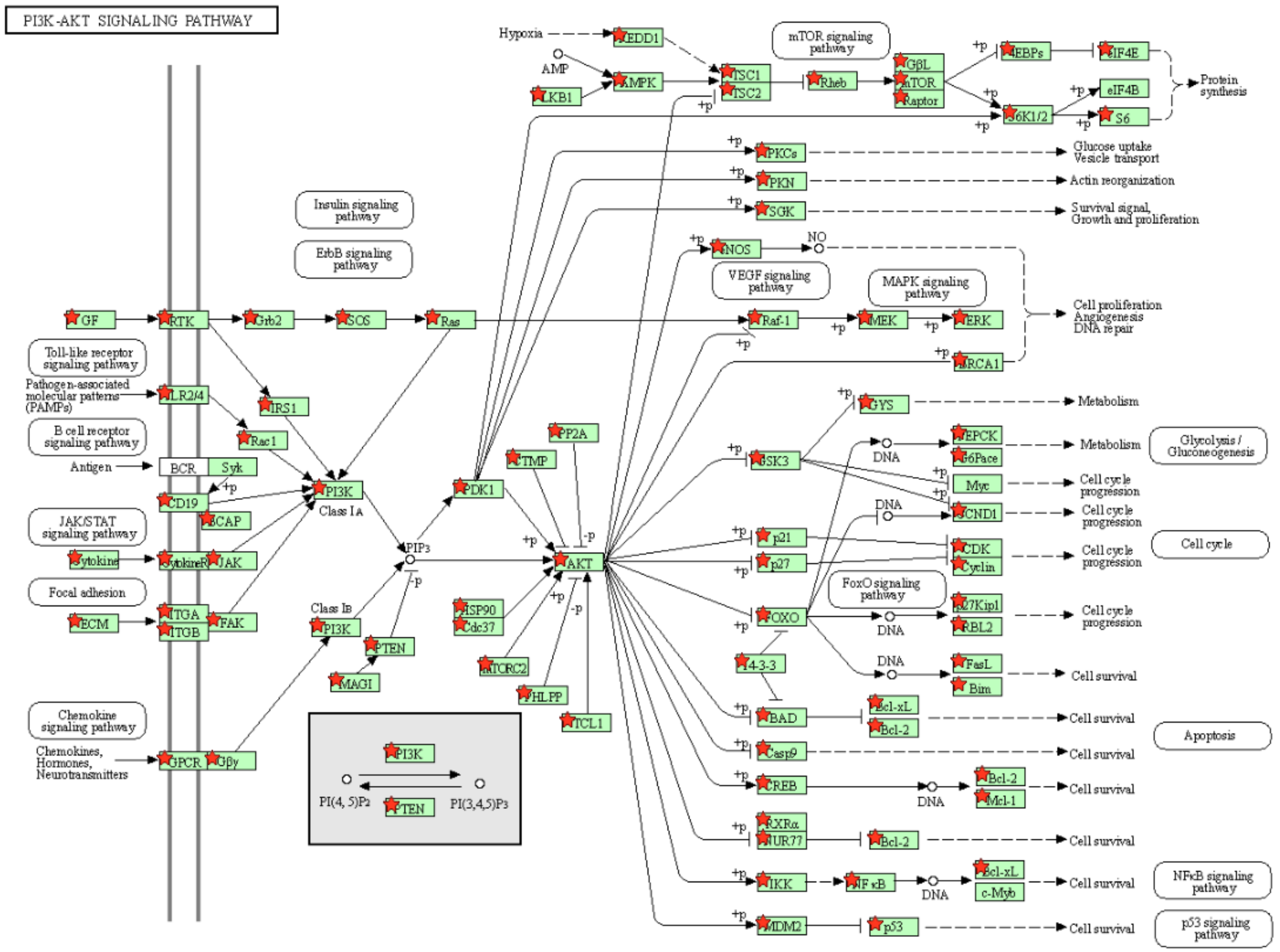

Supplementary Figure 1. The (downregulated) gene targets of upregulated miRNA impacted within the PI3K/Akt pathway determined by DAVID (v6.8) functional annotation analysis tool. Expression changes observed within FhHDM-1 treated β-cells under inflammatory conditions compared to controls treated with pro-inflammatory cytokines only. Activated genes are indicated by the red stars.

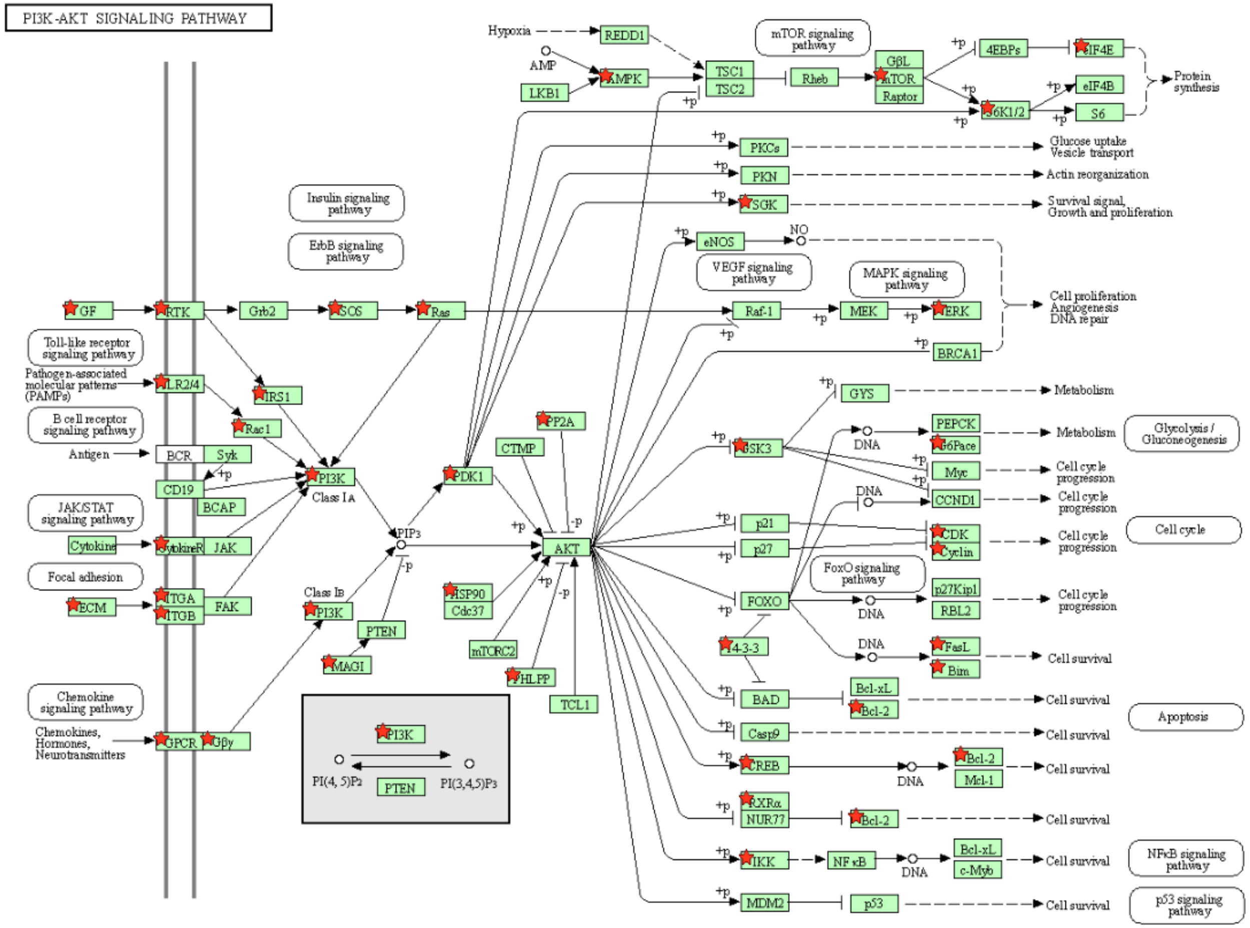

Supplementary Figure 2. The (upregulated) gene targets of downregulated miRNAs impacted within the PI3K/Akt pathway as determined using DAVID (v6.8) functional annotation analysis tool. Gene expression level changes observed within FhHDM-1 treated β-cells under inflammatory conditions as compared to control NIT-1 β-cells treated with pro-inflammatory cytokines only. Genes deemed to be increased in expression are indicated by the red stars
